# Discovery and characterization of chondroitinase A with specificity for 4-*O*-sulfated GalNAc in chondroitin sulfate/dermatan sulfate

**DOI:** 10.1016/j.jbc.2026.113594

**Published:** 2026-09-22

**Authors:** Wenshuang Wang, Haixin Yi, Ping’en Zi, Xu Wang, Rouyi Zou, Yu-zhong Zhang, Fuchuan Li

**Affiliations:** National Glycoengineering Research Center, State Key Laboratory of Microbial Technology, Shandong Center of Technology Innovation for Carbohydrate, Shandong Key Laboratory of Carbohydrate and Carbohydrate-conjugate Drugs, Shandong University, Qingdao, People's Republic of China

**Keywords:** chondroitin sulfate, lyase, substrate specificity, deep sea, glycosaminoglycan

## Abstract

Chondroitin sulfate (CS) and dermatan sulfate (DS) are glycosaminoglycans whose diverse biological functions are largely determined by specific sulfation patterns. The GlcA/IdoA-GalNAc4S (A/iA unit), where GlcA, IdoA, GalNAc and 4S denote D-glucuronic acid, L-iduronic acid, GalNAc, and 4-*O*-sulfation, respectively, is the most prevalent sulfation motif in animal tissues and participates in a broad spectrum of physiological and pathological processes. However, chondroitinase A (CSase A), a lyase capable of selectively degrading CS/DS rich in A/iA units, has not yet been identified, which has limited our understanding of the functional roles of such structures in CS/DS. Here, we report the first discovery of a CSase A from the metagenome of sediments near the Haima cold seep. This enzyme belongs to polysaccharide lyase family 8 and exhibits strict selectivity for A/iA units, yielding disaccharides composed almost exclusively of the 4-*O*-sulfated disaccharide, with no other sulfated disaccharide variants detectable even under exhaustive digestion. Structural analysis of resistant tetrasaccharides and hexasaccharides, and degradation assays with structure-defined oligosaccharides demonstrate that CSase A exclusively cleaves the β-1,4 glycosidic bond preceding a disaccharide unit containing GalNAc or GalNAc4S, thereby defining its rigorous substrate specificity. Site-directed mutagenesis and activity profiling identified Glu-579, Arg-480, Tyr-427, and His-420 as residues essential for catalysis, supporting a proposed tyrosine-histidine-mediated acid-base mechanism. The discovery of CSase A provides a unique molecular tool for dissecting the functional roles of A/iA units in CS/DS-related biology and disease.

Chondroitin sulfate (CS) and dermatan sulfate (DS, formerly known as CS-B) are sulfated linear polyanionic polysaccharides composed of repeating disaccharide units consisting of GalNAc and D-glucuronic acid (GlcA)/L-iduronic acid (IdoA). Alongside hyaluronic acid (HA), heparin/heparan sulfate (Hep/HS), and keratan sulfate, they constitute the four major groups of glycosaminoglycans (GAGs). As glycan side chains of proteoglycans, CS/DS are synthesized within the endoplasmic reticulum-Golgi system and are widely present on the surface and in the extracellular matrix of animal cells (1, 2, 3). A growing body of research has demonstrated that CS/DS are not only crucial structural components of tissues but also play pivotal roles in various physiological and pathological processes such as cell adhesion (4), migration (5), proliferation (6), differentiation (7, 8), tissue morphogenesis (9), angiogenesis modulation (10, 11), viral infection (12, 13), tumorigenesis (14), and cellular signaling (15, 16), through interactions with target proteins such as growth factors, chemokines, and collagens.

The remarkable functional specificity of CS/DS is fundamentally determined by their structural diversity, particularly with respect to sulfation patterns of GalNAc and/or GlcA/IdoA residues. The common CS chains primarily consist of six disaccharide units with distinct sulfation patterns, including GlcAβ1-3GalNAc (O unit), GlcAβ1-3GalNAc(4S) (A unit), GlcAβ1-3GalNAc(6S) (C unit), GlcA(2S)β1-3GalNAc(6S) (D unit), GlcAβ1-3GalNAc(4S,6S) (E unit), and GlcA(2S)β1-3GalNAc(4S,6S) (T unit), where 2S, 4S, and 6S denote 2-*O*-, 4-*O*-, and 6-*O*-sulfation, respectively. In contrast, with the exception of some marine animal-derived DS, the typical mammalian DS chains predominantly consist of three disaccharide units: IdoAα1-3GalNAc (iO unit), IdoAα1-3GalNAc(4S) (iA unit), and IdoA(2S)α1-3GalNAc(4S) (iB unit) (2). The distinct sulfation motifs confer unique biological activities by mediating selective interactions with various protein partners (17). Critically, among these structural variants, the 4-*O*-sulfated A/iA unit represents the most prevalent sulfation pattern. For example, CS-A isolated from mammalian cartilage contains more than 55% A units, while iA units account for approximately 90% of disaccharide residues in DS from mammalian skin and intestinal mucosa. These specific 4-*O*-sulfation patterns mediate crucial biological functions through selective molecular recognition mechanisms. The A unit-rich CS sequences serve as specific recognition sites for VAR2CSA protein in *Plasmodium falciparum*-infected erythrocytes, facilitating placental sequestration in malaria pathogenesis (18, 19, 20). Recent studies indicate that the specific CS-A-like structure recognized by VAR2CSA is overexpressed in a variety of tumors, positioning it as a potential pan-cancer diagnostic marker and a novel target for targeted therapy (21, 22, 23). Despite their high abundance and functional significance, research into A/iA unit-rich domains has been constrained by the absence of tools for their selective manipulation.

Microbial-derived chondroitinases (CSases) are essential tools for the structural and functional studies of CS/DS. As polysaccharide lyases, CSases cleave the β1-4 glycosidic linkage between GalNAc and GlcA/IdoA through a β-elimination reaction, generating an unsaturated double bond between the C4 and C5 positions of the uronic acid (24, 25, 26, 27). Based on substrate selectivity, identified CSases can be broadly classified into four major categories (28): CSase ABC, which degrades various CS and DS structures (29, 30, 31); CSase AC, which specifically degrades CS but not DS (32, 33, 34); CSase B, which specifically acts on DS (32); and CSase C, which selectively cleaves regions composed of C unit while exhibiting minimal activity toward A unit-rich domains (35, 36). To date, however, no enzyme capable of specifically degrading regions composed of 4-*O*-sulfated A/iA units, which would logically be termed CSase A, has been identified, leaving a conspicuous gap in this enzymatic toolkit. Given that these units represent the most abundant sulfation motif in terrestrial CS/DS, their functional study is a research priority. Therefore, the discovery and identification of a CSase A that can selectively degrade regions rich in 4-*O*-sulfated A/iA unit in CS/DS would provide a much-needed tool for various scientific and applied purposes, including investigations into the functions of A/iA unit in CS/DS chains, specific analysis and detection of A/iA unit and their constituent fragments in CS/DS samples, and the selective enzymatic preparation of functional oligosaccharides with other sulfation patterns in CS/DS chains.

In this study, we report the discovery and comprehensive characterization of the first authentic member of the CSase A subfamily, a long-postulated but previously uncharacterized class of CSases. Detailed enzymatic profiling demonstrates its unprecedented and strict specificity for cleaving glycosidic bonds that precede a disaccharide unit containing either GalNAc or GalNAc4S in CS/DS. This unique activity profile distinguishes CSase A from all known CSase classes (ABC, AC, B, and C) and establishes it as a novel enzymatic tool for the precise dissection of A/iA unit-rich functional domains. Our findings not only fill a critical gap in the GAG degradation toolkit but also provide a powerful molecular tool for deciphering A/iA unit-mediated biological functions and advancing the development of targeted glycotherapeutic strategies.

## Results

### Information of gene and protein sequences

The CSase A gene consists of 2856 base pairs with a GC content of 47.16%. It encodes a protein of 952 amino acid residues, exhibiting a predicted molecular mass of 107.97 kDa and an pI of 6.85. Sequence analysis *via* BLASTp identified an N-terminal signal peptide (Met1-Phe19), a 160-amino acid N-terminal region (Ala20-Thr179), and revealed the presence of a "GAG lyase superfamily module" (Lys215-Pro887), confirming its classification as a GAG lyase. The GAG lyase module contains four conserved candidate catalytic residues (His-420, Tyr-427, Arg-480, Glu-579 in CSase A without the N-terminal signal peptide) that are conserved among related GAG lyases. Phylogenetic analysis placed CSase A within the CSase ABC cluster, specifically belonging to the polysaccharide lyase family 8 (Fig. 1). Notably, CSase A shares the highest amino acid sequence similarity with the characterized CSase ABC I from *Proteus vulgaris* (P59807.2) among known enzymes, but this similarity level is relatively low (94% coverage, 29.72% identity).

**Figure 1:**
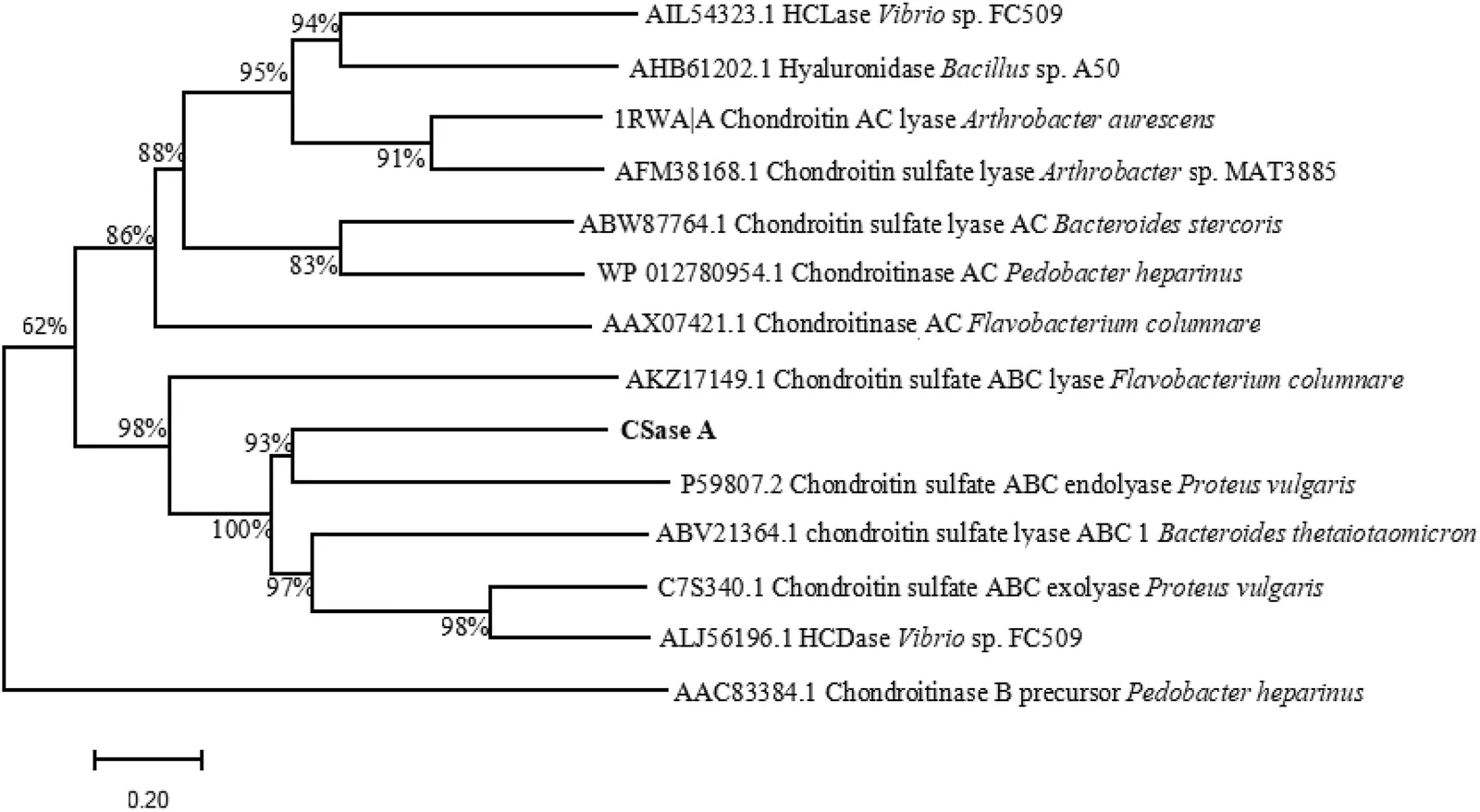
**Phylogenetic analysis of CSase A.** CSase A, chondroitinase A.

### Cloning, expression, and purification of rCSase A

The gene encoding the full-length CSase A without the N-terminal signal peptide was amplified from metagenomic DNA and cloned into the pET-30a(+) expression vector. The recombinant plasmid was transformed into *Escherichia coli* BL21(DE3) cells for heterologous expression. Following induction, cells were harvested, lysed by sonication, and the crude enzyme extract was obtained by centrifugation. The recombinant protein (rCSase A) was subsequently purified using Ni–NTA affinity chromatography. SDS–PAGE analysis confirmed the successful soluble expression of rCSase A, and the purified enzyme exhibited a purity exceeding 90%, with a recovery yield of approximately 75% and an initial protein concentration of 2 mg/ml (Fig. 2). These results demonstrate an efficient expression and purification process, providing a sufficient amount of active enzyme for subsequent biochemical characterization.

**Figure 2:**
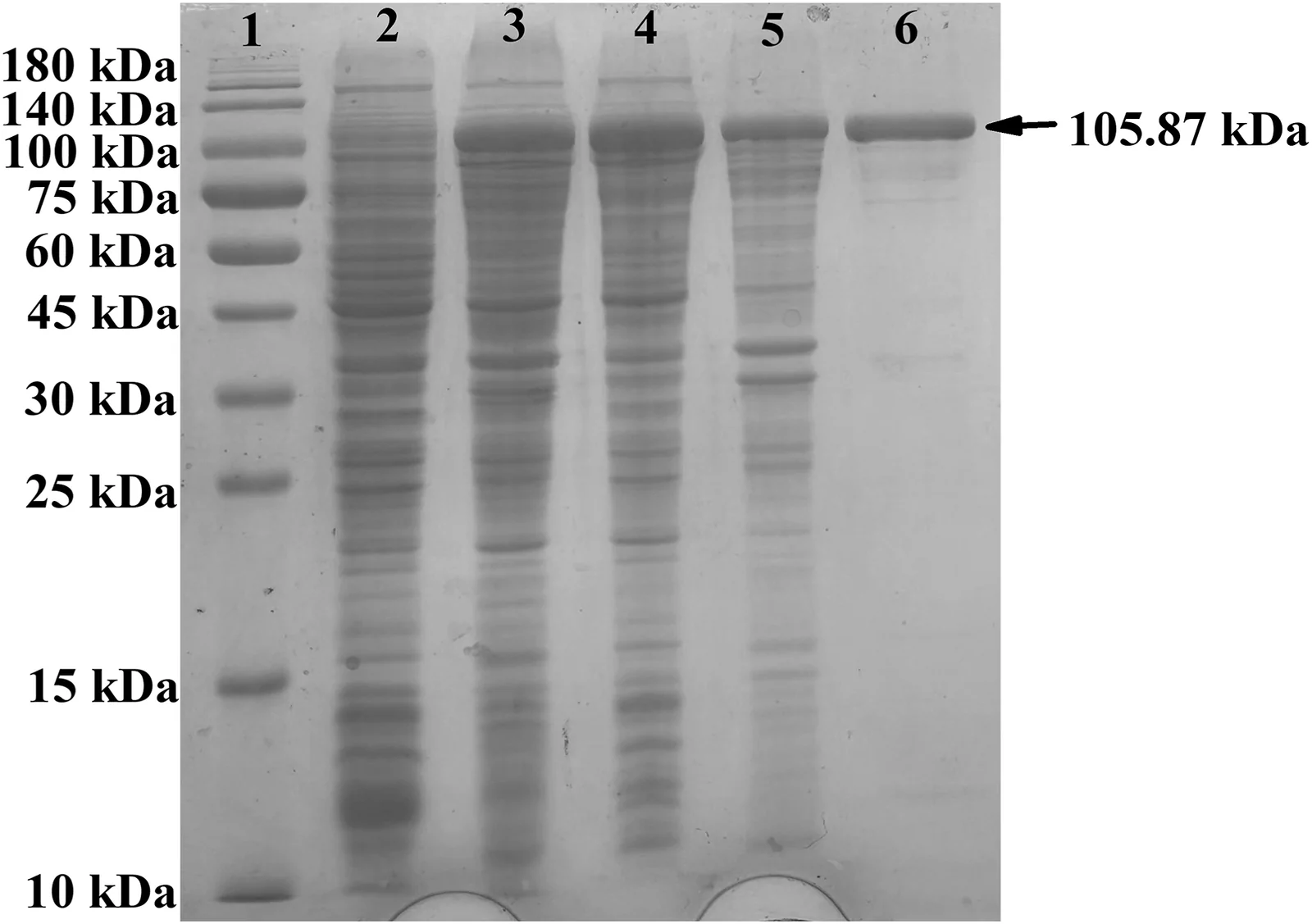
**Expression and purification profiles of CSase A.** Recombinant CSase A was expressed, purified, and analyzed by SDS-PAGE. *Lane 1*, prestained protein molecular marker PageRuler (26,616, Thermo Fisher Scientific); *lane 2*, cell lysate of transformed empty plasmid (pET-30a); *lane 3*, cell lysate after CSase A expression; *lane 4*, supernatant of cell lysate after CSase A expression; *lane 5*, precipitation of cell lysate after CSase A expression; *lane 6*, purified target protein CSase A. Molecular weight marker and corresponding molecular weight of the protein were labeled. CSase A, chondroitinase A.

### Substrate selectivity of rCSase A

To assess the substrate selectivity of rCSase A, various GAGs and their analogs were incubated with the purified enzyme under standard conditions, and the resulting products were analyzed by ultraviolet spectrophotometer and gel filtration chromatography. Enzyme activity toward different substrates was initially assessed by monitoring the increase in absorbance at 232 nm, which reflects the formation of an unsaturated double bond between C4 and C5 of the hexuronic acid at the nonreducing end of the oligosaccharide products generated *via* a lyase-catalyzed β-elimination mechanism. rCSase A exhibited activity toward DS, HA, and various CS substrates but showed no significant activity toward Hep, HS or alginate (Alg), suggesting that rCSase A belongs to the CSase ABC-type enzyme family.

### Enzymatic characteristics of rCSase A

Using CS-A as representative substrate, the optimal reaction conditions for rCSase A were systematically evaluated. Maximum enzymatic activity was observed at 10 °C for CS-A (Fig. 3*A*), suggesting that the enzyme is cold-adapted, consistent with its origin from deep-sea Haima cold seep sediments. rCSase A exhibited maximal activity toward CS-A in 50 mM phosphate buffer at pH 6.0 (Fig. 3*B*). However, in acetate buffer at the same pH, its activity was only one-fourth of that observed in phosphate buffer. Interestingly, within the acetate buffer system, the enzyme displayed higher activity at pH 5 than at pH 6. This buffer-dependent difference in activity may stem from the influence of different buffer ions on the surface charge distribution or the conformation of the active site, suggesting that phosphate ions might contribute to maintaining the structural state necessary for catalysis. This contrasts with *P. vulgaris* CSase ABC I, which demonstrates a preference for Tris-HCl buffer over phosphate buffers (30, 37). When CS-C and HA were used as substrates under the same buffer conditions (Fig. 3*B*), low but measurable activities were observed, with pH-dependent profiles broadly similar to that of CS-A but at substantially lower levels. Importantly, HA-degrading activity was not selectively enhanced under acidic conditions, indicating that rCSase A does not exhibit the low-pH-dependent hyaluronidase activity reported for conventional CSase ABC. The detectable activity toward commercial CS-C may mainly reflect cleavage of its intrinsic A-unit-containing sequences rather than C units themselves. Metal ion screening identified potent inhibitors including Ag^+^, Co^2+^, Cu^2+^, Ni^2+^, Hg^2+^, Pb^2+^, Zn^2+^, and Fe^3+^ (Fig. 3*C*). Notably, CSase A activity remained unaffected by NaCl, a key distinction from the NaCl-sensitive *P*. *vulgaris* CSase ABC I (37). Thermostability studies revealed that rCSase A was quite stable and could retain 90% activity for at least 24 h at temperatures between 0 °C and 20 °C. At 30 °C and 40 °C, the activity exhibited a time-dependent decrease, dropping to approximately 50% after 8 h and further declining to about 20% by 24 h. In contrast, at 50 °C, 60 °C, and 70 °C, a more gradual but sustained decline was observed over an extended period. Although activity decreased within the first hour (to approximately 70%, 55%, and 45%, respectively), it continued to diminish progressively, eventually reaching near-zero levels only after approximately 15 to 20 h (Fig. 3*D*). Freeze-thaw stability assays showed that rCSase A retained more than 80% of its initial activity after three cycles and approximately 50% after six cycles (Fig. 3*E*), indicating reasonable tolerance to repeated freeze-thaw handling. To identify MS-compatible reaction conditions, rCSase A activity was further evaluated at pH 6.0 and 10 °C either without an added buffer or in 50 mM ammonium acetate buffer (pH 6.0). More than 70% of the activity observed in phosphate buffer was retained under both conditions, and more than 90% was retained in ammonium acetate buffer, demonstrating that this volatile buffer provides an effective MS-compatible alternative with only a minor loss of activity (Fig. 3*F*). Collectively, these findings reveal that rCSase A possesses a unique biochemical profile distinct from that of *P. vulgaris* CSase ABC I. Based on these optimized conditions, all subsequent reactions were carried out at 10 °C in 50 mM NaH_2_PO_4_-Na_2_HPO_4_ buffer (pH 6.0).

**Figure 3:**
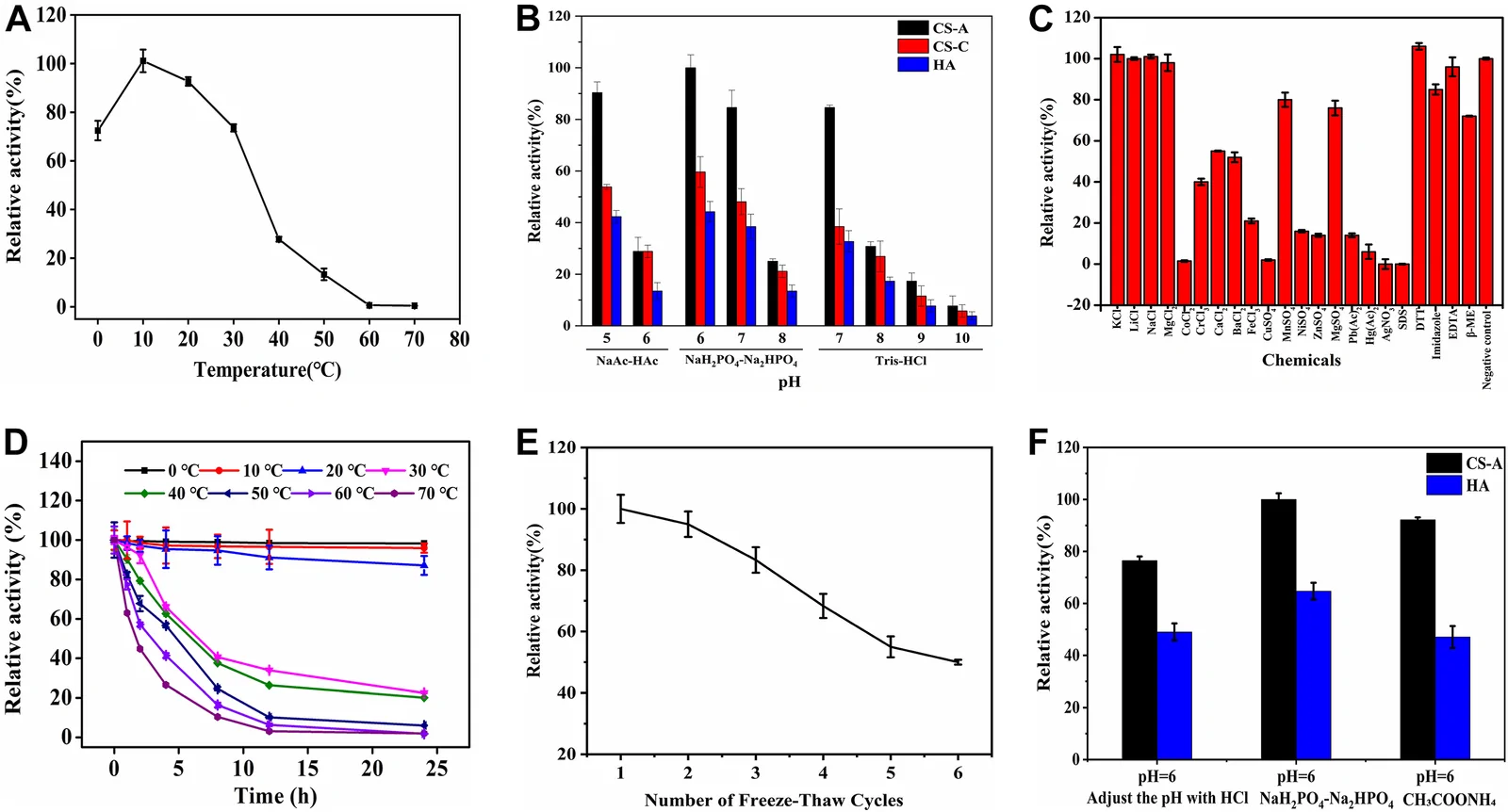
**Biochemical characterization of CSase A.***A*, effect of temperature. The enzyme activity of CSase A was assayed in 50 mM NaH_2_PO_4_-Na_2_HPO_4_ (pH 7.0) buffer at different temperatures (0 °C-70 °C) with CS-A as substrates. Readings at the optimum temperature are shown as 100% and data for the remaining temperatures are shown as relative percentages of values obtained at the optimum temperature. *B*, effect of pH on enzyme activity. Effect of pH on enzyme activity. The activity of rCSase A toward CS-A, CS-C, and HA was assayed at the optimal temperature in buffers of different pH values (50 mM NaAc-HAc, pH 5.0–6.0; 50 mM NaH_2_PO_4_-Na_2_HPO_4_, pH 6.0–8.0; 50 mM Tris-HCl, pH 7.0–10.0). The activity of CS-A at 50 mM NaH_2_PO_4_-Na_2_HPO_4_ pH 6.0 was defined as 100%, and the relative activities at other pH values for all three substrates were calculated accordingly. *C*, effect of metal ions and chelating agents. The effect of different metal ions and chelating agents (5 mM) on the degradation of CS-A by CSase A was examined at its optimum temperature and optimum pH. The data shown in the graphs are based on the values obtained in buffer without metal ions or chelating agents as 100%, and the rest of the values are the relative percentage of that control value. *D*, thermostability. The enzyme was incubated at different temperatures (0 °C-70 °C) for different times (0–24 h), and the residual enzyme activity against CS-A was assayed under optimal conditions for CSase A. The reading of the unincubated enzyme was taken as 100% and the value after incubation was taken as the relative percentage of that value. *E*, freeze-thaw stability. rCSase A was subjected to repeated freeze-thaw cycles, and residual activity was measured after each cycle. *F*, activity in MS-compatible buffer. The activity of rCSase A toward CS-A was evaluated at pH 6.0 and the optimal temperature under three conditions: 50 mM phosphate buffer (control), direct pH adjustment of the substrate solution to pH 6.0 without buffering, and 50 mM ammonium acetate buffer (pH 6.0). The error bars are the mean ± S.D. values of three parallel sets of experimental groups. CS, chondroitin sulfate; CSase A, chondroitinase A; HA, hyaluronic acid.

Under these optimized conditions, the specific activity of rCSase A was determined using HA, DS, and DS from *Halocynthia roretzi* (HRDS), and several commercial CS variants (Table 1). The enzyme displayed the highest activity toward CS-A (612.51 mU/mg), followed by DS (510.31 mU/mg), HRDS (57.99 mU/mg), CS-C (43.63 mU/mg), CS-E (38.05 mU/mg), CS-D (23.46 mU/mg), and HA (7.86 mU/mg). To relate this activity profile to substrate structure, the disaccharide composition of each CS/DS substrate was analyzed (Table S1). CS-A and DS, the preferred substrates, were enriched in A/iA units (81.68% A units in CS-A and 85.71% A/iA units in DS). In contrast, the other CS/DS preparations were enriched in alternative sulfated disaccharide units, whereas HA consisted entirely of unsulfated disaccharides. Notably, HRDS, which shares the dermatan backbone with DS but contains 74.25% iB units (IdoA2S1-3GalNAc4S) and only 22.35% iA units, exhibited substantially lower activity than conventional DS, supporting the conclusion that 2-*O*-sulfation of the uronic acid residue strongly restricts cleavage. Collectively, these data indicate that rCSase A preferentially degrades A/iA-rich CS/DS structures and is highly sensitive to sulfation at other positions. These observations prompted us to further define its substrate specificity using structurally defined oligosaccharides.

**Table 1:** Enzyme activity of CSase A on different substrates

Substrates	Activity of CSase A
	mU/mg
CS-A	612.51 ± 1.07
CS-C	43.63 ± 0.51
CS-D	23.46 ± 0.63
CS-E	38.05 ± 1.05
DS	510.31 ± 1.07
HRDS	57.99 ± 1.03
HA	7.86 ± 0.61

### Substrate-degrading pattern of CSase A

To elucidate the substrate-degrading pattern of CSase A, a time-course digestion experiment was performed using CS-A (1 mg) as the substrate at 10 °C in 50 mM NaH_2_PO_4_-Na_2_HPO_4_ buffer (pH 6.0), with fresh enzyme added at 12 h to sustain sufficiently high activity. Analysis of the digestion products by gel-filtration chromatography on a Superdex peptide 10/300 Gl column (monitored at 232 nm) revealed a progressive shift in molecular size distribution. Higher molecular weight oligosaccharides predominated in the early stages, followed by the progressive accumulation of smaller oligosaccharides (Fig. 4). This sequential substrate depolymerization, from larger fragments to smaller unsaturated oligosaccharides, confirms that rCSase A functions as an endo-type CS/DS lyase that cleaves polysaccharide chains internally in a random manner, rather than sequentially from the chain termini. Compositional analysis of the disaccharide products collected at different time points further revealed that ΔA units (ΔHexA1-3GalNAc4S, where ΔHexA represents an unsaturated hexuronic acid) were overwhelmingly dominant at all time points examined, and ΔO units (ΔHexA1-3GalNAc) (0.52–6.94%) began to appear after 4 h (Table S3), whereas ΔC units (ΔHexA1-3GalNAc6S) were not detected throughout the entire digestion period. This finding preliminarily suggested that the enzyme preferentially cleaves A/iA units during CS-A degradation.

**Figure 4:**
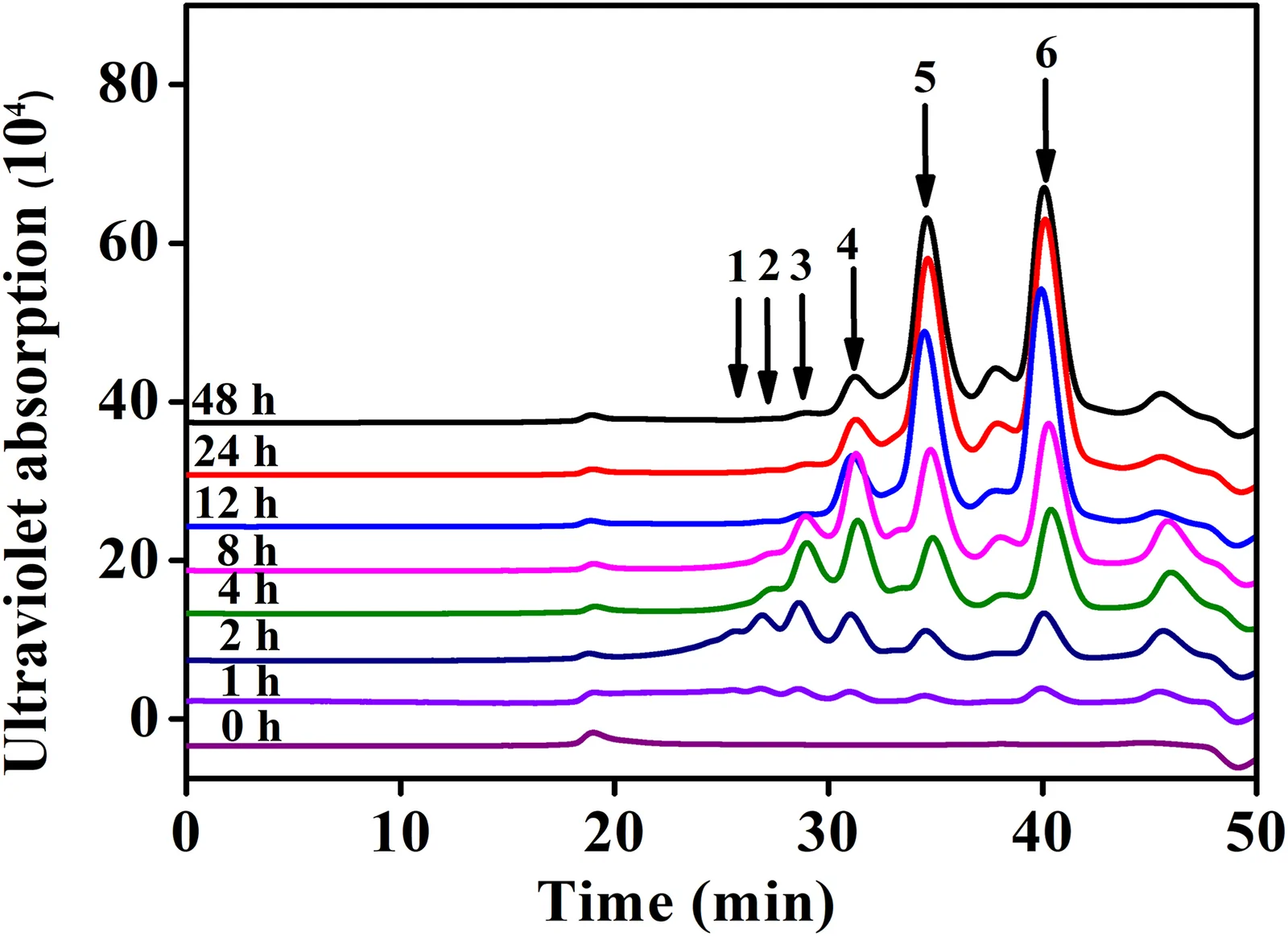
**Time-course digestion of CS-A by CSase A.** CS-A (1 mg) was digested with 100 μg of CSase A, and aliquots were taken at the indicated time points. Reactions were terminated by heat inactivation. A portion (10 μg) of each time-point sample was analyzed by gel-filtration chromatography. The elution positions of CS-A degradation end-products are indicated by the *arrows*: 1, unsaturated dodecasaccharide; 2, unsaturated decasaccharide; 3, unsaturated octasaccharide; 4, unsaturated hexasaccharide; 5, unsaturated tetrasaccharide; 6, unsaturated monosulfated disaccharide. CS, chondroitin sulfate; CSase A, chondroitinase A.

To further investigate its substrate selectivity of CSase A, residual tetrasaccharide and hexasaccharide fractions that remained undegraded after exhaustive digestion were collected and subjected to disaccharide composition analysis. The results were compared with those of the disaccharide products generated by complete digestion. In the disaccharide fraction, ΔA units predominated, accounting for 93.06% of the total, while ΔC units were undetectable. In contrast, the residual tetrasaccharide and hexasaccharide fractions contained markedly lower proportions of ΔA units (37.53% and 11.79%, respectively). From disaccharide to tetrasaccharide to hexasaccharide, the proportion of ΔA units exhibited a stepwise decrease, whereas ΔC units, which were absent in the disaccharide fraction, increased stepwise to 62.47% in the tetrasaccharide fraction and further to 88.21% in the hexasaccharide fraction (Table 2). These results strongly indicate that CSase A selectively avoids cleaving domains containing ΔC units, thereby providing robust evidence for its strict preference for A/iA units.

**Table 2:** Analysis of disaccharide composition of oligosaccharides collected after degradation of CS-A by CSase A

Oligosaccharide	ΔO	ΔC	ΔA	ΔB	ΔD	ΔE
	Mol %					
disaccharide	6.94	ND	93.06	ND	ND	ND
tetrasaccharide	ND	62.74	37.53	ND	ND	ND
hexasaccharide	ND	88.21	11.79	ND	ND	ND

Mol %: The molar ratio of disaccharide units was calculated based on their peak areas.

ND: not detected.

On this basis, we expanded the substrate scope to include HA, DS, and various CS isoforms. Each substrate was subjected to exhaustive digestion under identical conditions, and the products were analyzed by gel-filtration chromatography. The results demonstrated that the degradation products from different substrates consisted of oligosaccharides with varying degrees of polymerization (Fig. 5). Notably, during the digestion of sulfated CS/DS substrates, the predominant disaccharide products were monosulfated disaccharides, contrasting with the heterogeneous disaccharide compositions of the original substrates (Table S1). To further explore this phenomenon, the collected disaccharides were subjected to detailed compositional analysis by anion-exchange HPLC. As shown in the corresponding inset panels (Fig. 5, *A–E*) and Table S2, despite the heterogeneous sulfation patterns of the starting CS/DS substrates, ΔA units were overwhelmingly dominant in the disaccharide product profiles. This unique product profile provides compelling evidence for the pronounced substrate preference of rCSase A, namely, its preferential cleavage of glycosidic bonds adjacent to GalNAc4S residues.

**Figure 5:**
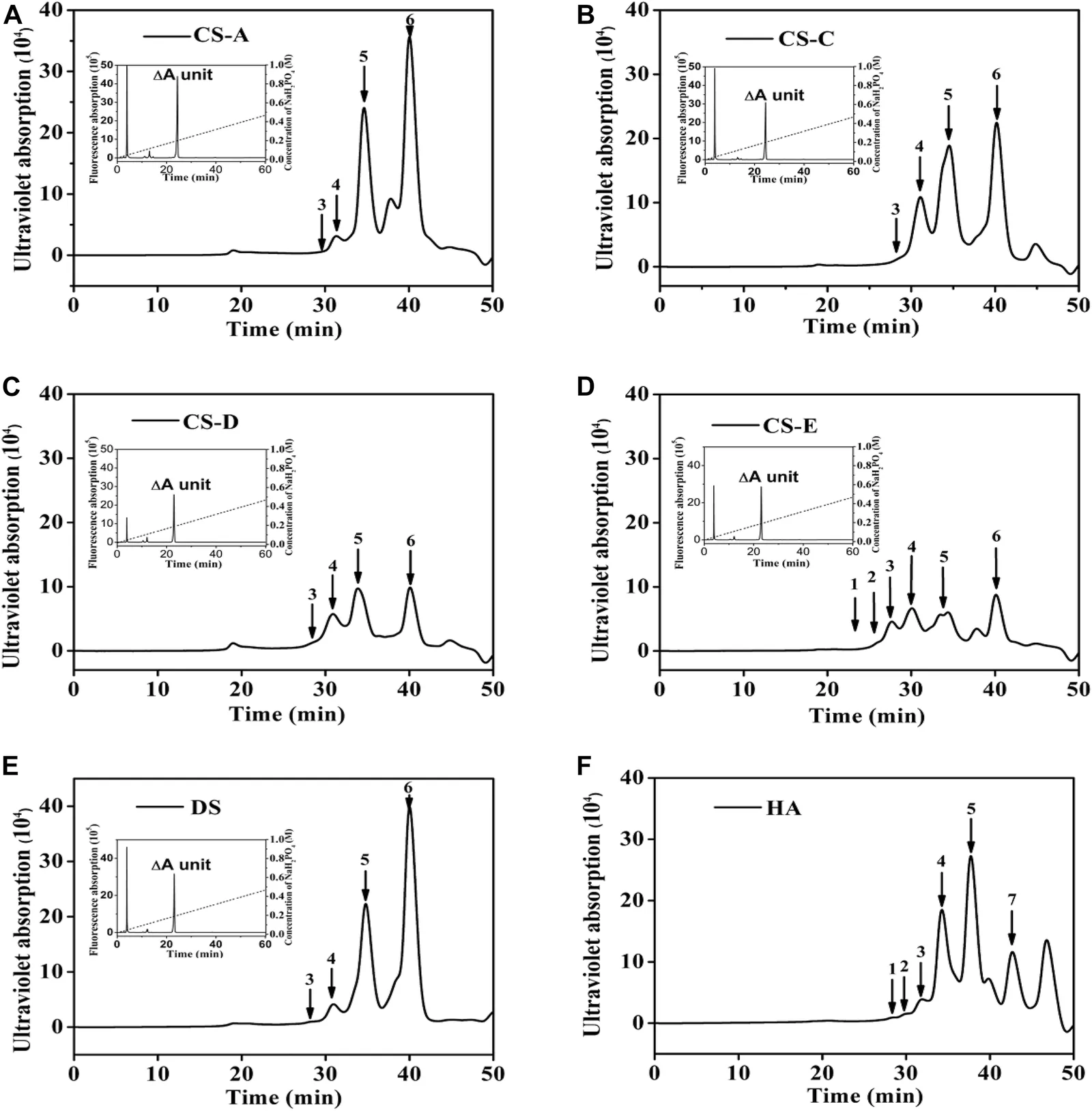
**Analysis of products from exhaustive degradation by CSase A.** CS-A (*A*), CS-C (*B*), CS-D (*C*), CS-E (*D*), DS (*E*), and HA (*F*) were exhaustively digested with CSase A. The digests were analyzed by gel-filtration chromatography. The elution positions of the oligosaccharides are indicated by *arrows*: 1, unsaturated dodecasaccharide; 2, unsaturated decasaccharide; 3, unsaturated octasaccharide; 4, unsaturated hexasaccharide; 5, unsaturated tetrasaccharide; 6, unsaturated monosulfated disaccharide; 7, unsaturated nonsulfated disaccharide. The disaccharide fractions collected from each digest were further subjected to compositional analysis by anion-exchange HPLC, and the results are shown in the insets. CS, chondroitin sulfate; CSase A, chondroitinase A; DS, dermatan sulfate; HA, hyaluronic acid.

In summary, this distinctive substrate selectivity distinguishes rCSase A from all previously characterized CSases and supports its designation as the first identified member of the CSase A family.

### Sequence analysis of resistant tetrasaccharides and hexasaccharides

To further investigate the structural determinants conferring resistance to CSase A, the resistant tetrasaccharide and hexasaccharide fractions obtained from CS-A digestion was isolated for subsequent sequence and structural analysis. The resistant oligosaccharide fractions were first separated by gel filtration as described in the “Experimental procedures”, followed by further purification by anion-exchange chromatography on a YMC-Pack PA-G column (Figs. S1 and S3). Two major subfractions were obtained from the resistant tetrasaccharide fraction (tetrasaccharide fractions 1 and 2), and two major subfractions were obtained from the resistant hexasaccharide fraction (hexasaccharide fractions 1 and 2). All were analyzed using an established enzymatic sequencing method (26, 38).Structural analysis revealed that Fractions 1 and 2 corresponded to ΔC-C and ΔA-C, respectively (Fig. 6). These tetrasaccharide structures were further confirmed by mass spectrometry. Unsaturated disulfated ΔC-C or ΔA-C was assigned to m/z 458.0468 and 458.0482 ([M-2H]^2-^), and m/z 939.0764 and 939.0775 ([M-H]^-^), respectively, with the observed m/z values matching the theoretical masses of these CSase A-resistant tetrasaccharides (Fig. S2). In addition, hexasaccharide fractions 1 and 2 were identified as ΔA-C-C and ΔC-C-C, respectively, using the same enzymatic sequencing method (Fig. 7). The structural features of these resistant oligosaccharides provide crucial insights into the catalytic mechanism of CSase A. Both tetrasaccharides and hexasaccharides share a common characteristic: the presence of GlcA-GalNAc6S at the reducing end. This conserved structural motif suggests that 6-*O*-sulfation at the reducing end creates a steric or electronic barrier to efficient cleavage by CSase A. Conversely, the enzyme demonstrates remarkable efficiency in cleaving bonds where the reducing end is occupied by either GlcA-GalNAc or GlcA-GalNAc4S residues.

**Figure 6:**
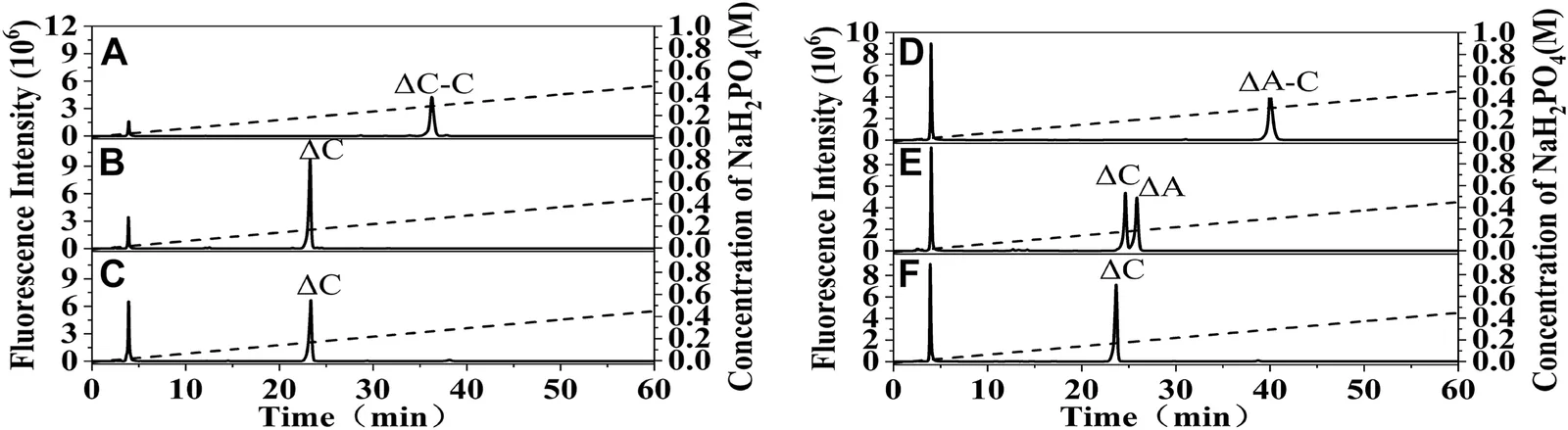
**Sequencing of resistant tetrasaccharides from CSase A-degraded CS-A.***A*, resistant tetrasaccharide 1, the resistant tetrasaccharide was first labeled with 2-AB and then subjected to anion-exchange column analysis; *B*, disaccharide composition analysis of resistant tetrasaccharide 1, the resistant tetrasaccharide was degraded with CSase ABC and analyzed using ion-exchange chromatography after 2-AB labeling; *C*, reducing-end disaccharides analysis of resistant tetrasaccharide 1, labeled resistant tetrasaccharide degraded using the exo-type HCDLase; *D*, resistant tetrasaccharide 2; *E*, disaccharide composition analysis of resistant tetrasaccharide 2; *F*, the reducing-end disaccharide analysis of resistant tetrasaccharide 2. 2-AB, 2-aminobenzamide; CS, chondroitin sulfate; CSase A, chondroitinase A; HCDLase, hyaluronan/chondroitin sulfate/dermatan sulfate lyase.

**Figure 7:**
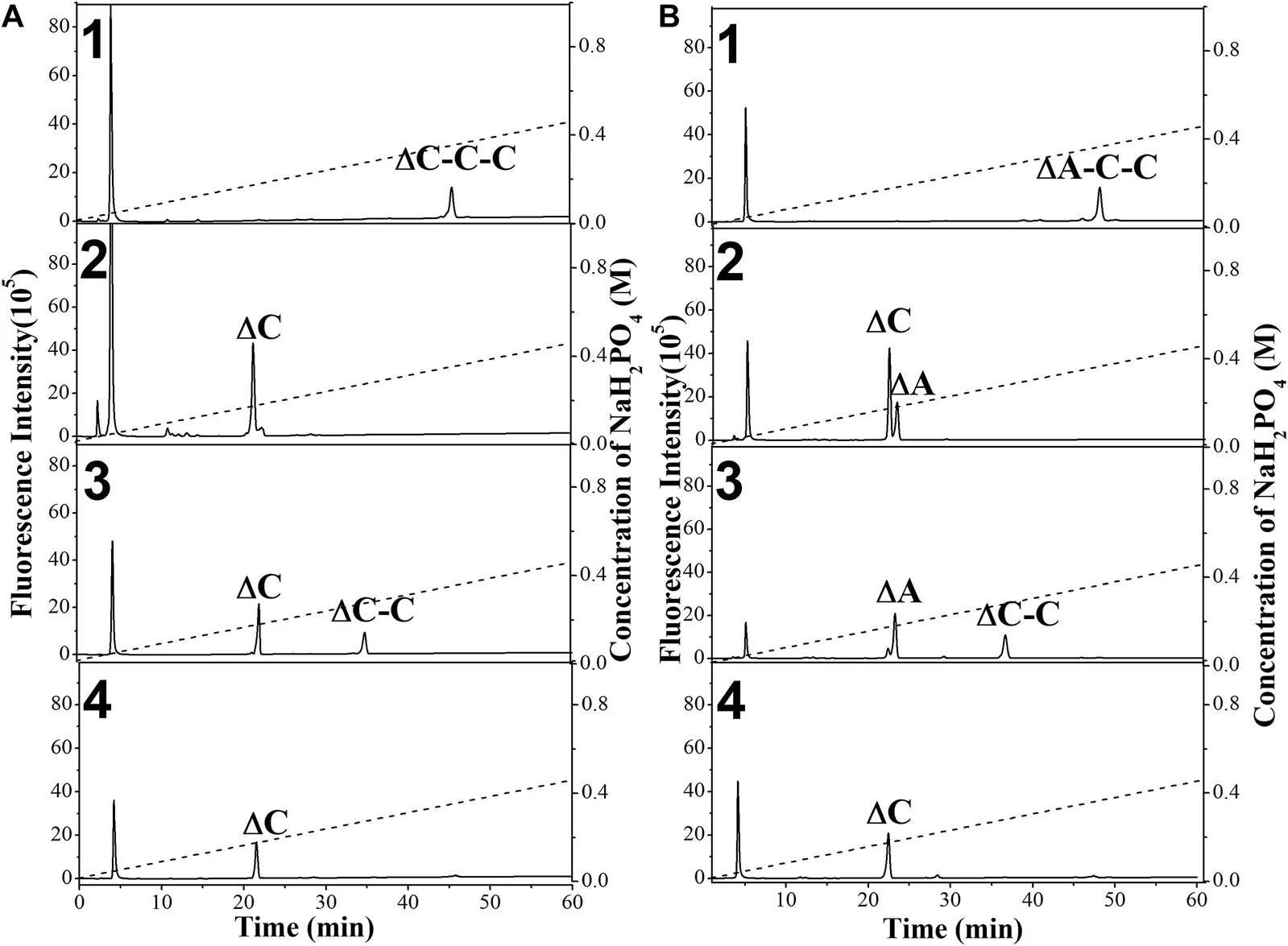
**Sequencing of resistant hexasaccharide from CSase A-degraded CS-A.***A*, resistant hexasaccharide 1; *B*, resistant hexasaccharide 2. 1, the resistant hexasaccharides were first labeled with 2-AB and then subjected to anion-exchange column analysis; 2, the disaccharide composition analysis of resistant hexasaccharides; 3, nonreducing-end disaccharides analysis of labeled resistant hexasaccharides using CSase ABC and then relabeled. 2-AB, 2-aminobenzamide; CS, chondroitin sulfate; CSase A, chondroitinase A.

### Assessment of degradation capacity toward different tetrasaccharide substrates

Building on the finding that CSase A cannot degrade tetrasaccharides with a C unit at the reducing end, we sought to determine whether this enzyme exhibits absolute specificity for A unit or is inactive toward 6-*O*-sulfation patterns. To address this question, a panel of defined unsaturated tetrasaccharide standards, ΔD-A (ΔD, ΔHexA2S1-3GalNAc6S), ΔE-A (ΔE, ΔHexA1-3GalNAc(4S,6S)), ΔA-D, ΔE-C, ΔE-E, and ΔB-iB (ΔHexA2S1-3GalNAc4S1-4IdoA2S1-3GalNAc4S), were used as substrates for detailed degradation analysis. The reaction products were systematically analyzed using sensitive anion-exchange chromatography after appropriate labeling. The results provide clear evidence for the strict substrate requirements of CSase A. The enzyme failed to degrade tetrasaccharides containing C unit, D unit, E unit, or iB unit at the reducing end (Fig. 8, *C–F*), while efficiently cleaving those bearing a 4-*O*-sulfated A unit at the same position (Fig. 8, *A* and *B*). This result indicates that specific sulfation modifications on the reducing-end disaccharide, particularly 6-*O*-sulfation of GalNAc residues or 2-*O*-sulfation of uronic acid residues, create structural impediments that prevent productive enzyme-substrate interaction. Notably, sulfation at the nonreducing end demonstrated no inhibitory effect, underscoring the enzyme’s selective sensitivity to reducing-end structural features. To determine whether 4-*O*-sulfation of the reducing-end GalNAc residue is required for cleavage, two tetrasaccharides bearing an A unit at the reducing end (ΔC-A and ΔD-A) were first treated with a 4-*O*-sulfatase to remove the 4-*O*-sulfate group, generating ΔC-O and ΔD-O, respectively. Both desulfated products were subsequently cleaved by CSase A to disaccharides (Fig. 8, *G* and *H*), demonstrating that 4-*O*-sulfation at the reducing end is not an absolute requirement for catalysis. To further test a nonsulfated polymeric substrate, chondroitin was incubated with CSase A and analyzed by size-exclusion chromatography. CSase A efficiently degraded chondroitin, with the nonsulfated disaccharide O unit as the major product (Fig. S4). These results indicate that rCSase A can accommodate either 4-*O*-sulfated or unsulfated GalNAc at the reducing end, whereas 6-*O*-sulfation of GalNAc or 2-*O*-sulfation of the uronic acid strongly restricts cleavage.

**Figure 8:**
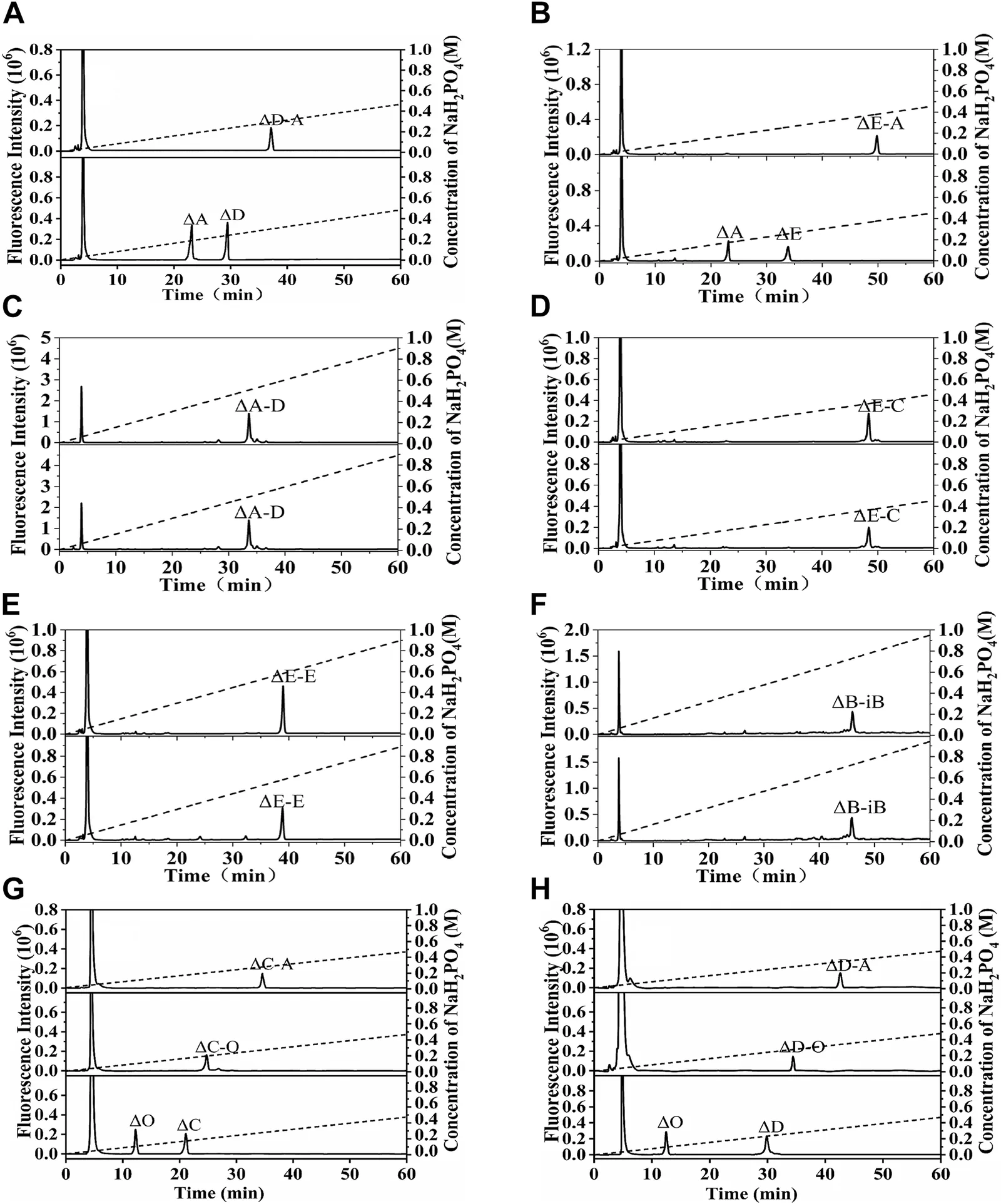
**Analysis of CSase A degradation products of standard tetrasaccharides.** Standard tetrasaccharides: ΔD-A (*A*), ΔE-A (*B*), ΔA-D (*C*), ΔE-C (*D*), ΔE-E (*E*), and ΔB-iB (*F*) were degraded with CSase A, labeled with 2-AB, and then analyzed by HPLC on a YMC Pack PA-G column. In each panel (*A–F*), the *upper graphs* show the analysis without enzymatic degradation, and the *bottom graphs* show the analysis after CSase A degradation. To further determine whether 4-*O*-sulfation at the reducing end is the sole determinant of resistance, ΔC-A (*G*) and ΔD-A (*H*) were first analyzed intact (*upper graphs*), then treated with 4-*O*-sulfatase to remove the 4-*O*-sulfate group from the reducing-terminal GalNAc (*middle graphs*), and the desulfated products were subsequently incubated with CSase A (*lower graphs*). CSase A, chondroitinase A; PA-G, polyamine guard.

This substrate specificity profile firmly establishes CSase A's unique position within the CSase family. Whereas CSase ABC enzymes display broad substrate tolerance and CSase C specifically targets 6-*O*-sulfated regions, CSase A shows strict specificity for 4-*O*-sulfated or nonsulfated motifs at the reducing end. This fundamental distinction not only validates its classification as the prototype of the CSase A subfamily but also highlights its unique functional specialization among GAG-degrading enzymes. Given that GalNAc4S represents the most abundant sulfation pattern in mammalian tissues, CSase A provides an unprecedented tool for dissecting the functional roles of these predominant structures. By enabling selective cleavage of A unit-rich domains while leaving other sulfation motifs intact, the enzyme allows researchers to isolate and characterize specific functional elements within complex GAG chains.

### Structural modeling and mutational analysis of CSase A

To elucidate the molecular basis of the catalytic mechanism of CSase A, a three-dimensional structural model was constructed using SWISS-MODEL with the structure of CSase ABC I from *P. vulgaris* (P59807.2) as a template (39). Both enzymes share a conserved architecture comprising N-terminal, central catalytic, and C-terminal domains (Fig. 9). However, CSase A lacks an α-helical segment within its central catalytic domain, a structural distinction that may be linked to its substrate selectivity. Given the low sequence identity (29.72%) between these two enzymes, we hypothesize that CSase A's distinct degradation specificity arises from this structural deletion and/or divergent amino acid composition, and this hypothesis needs further investigation.

**Figure 9:**
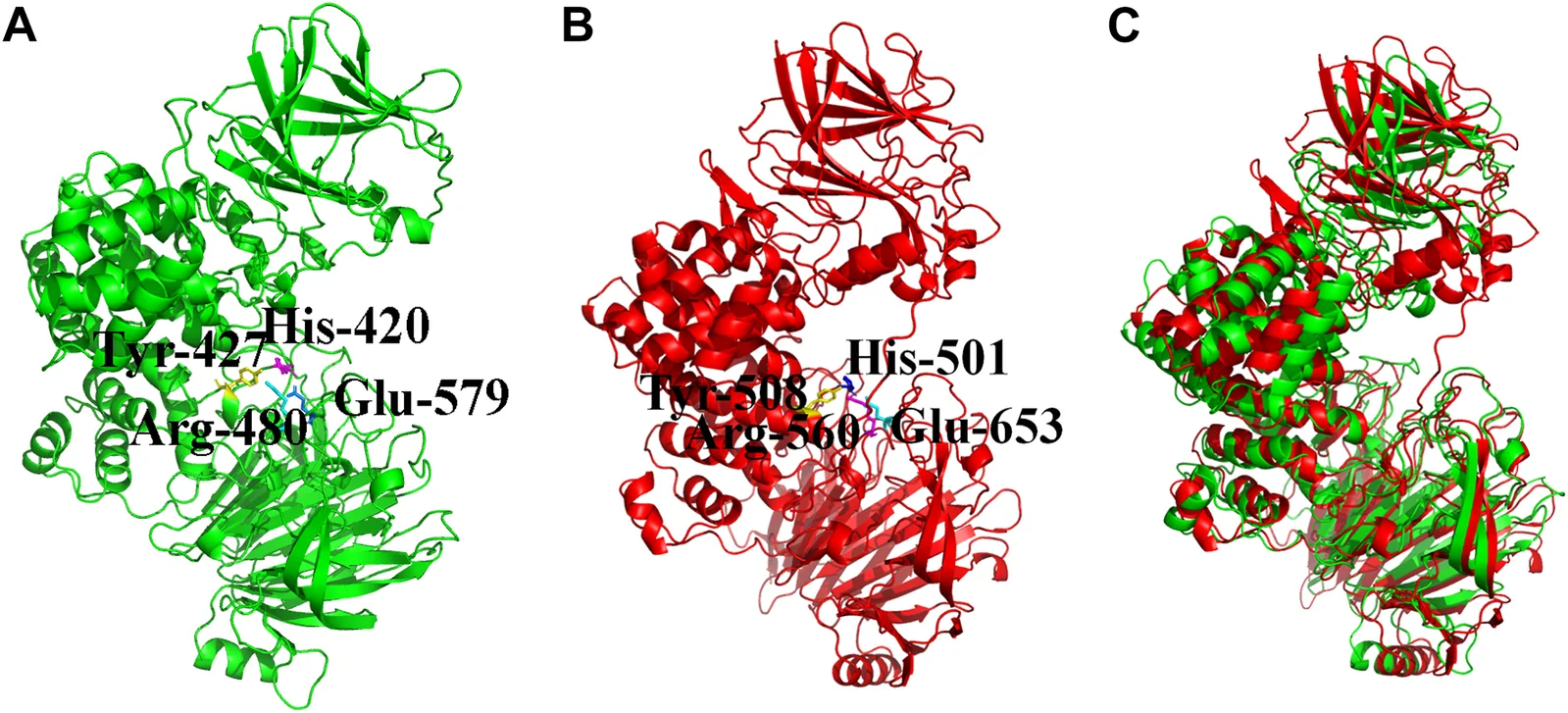
**Analysis of the tertiary structure of the CSase A.***A*, tertiary structure of CSase A (SWISS-MODEL online prediction); *B*, tertiary structure of CSase ABC I (P59807.2) from *Proteus vulgaris*; *C*, Structural comparison of CSase A and CSase ABC I. The model of CSase ABC I is shown in *red*, and that of CSase A is shown in *green*. Key amino acid sites are labeled in figures *A* and *B*. CSase A, chondroitinase A.

To preliminarily investigate the action mechanism of CSase A, four conserved amino acid residues His-420, Tyr-427, Arg-480 and Glu-579 were individually replaced by alanine using site-directed mutagenesis. Analysis of the mutants’ degradation capacity toward HA and CS by gel-filtration chromatography revealed distinct functional contributions. As shown in Figure 10, the CSase A-H420A mutant lost the ability to degrade both CS-A and DS, indicating that His-420 is essential for catalysis. CSase A-E579A and CSase A-R480A mutants showed partial inactivation against these substrates, suggesting supportive roles in substrate alignment or transition-state stabilization. Interestingly, CSase A-Y427A mutant lost all activity toward CS-A but retained partial degradation activity toward DS, implying that Tyr-427 contributes to substrate-recognition specificity (Fig. 10). These findings demonstrate that His-420, Tyr-427, Arg-480, and Glu-579 are essential for CSase A catalysis. Although their relative contributions differ between substrates, these four conserved residues likely stabilize the enzyme structure and/or participate directly in the acid-base catalytic mechanism during glycosidic bond cleavage (30). Collectively, these residues play indispensable roles in the substrate-cleavage activity of CSase A.

**Figure 10:**
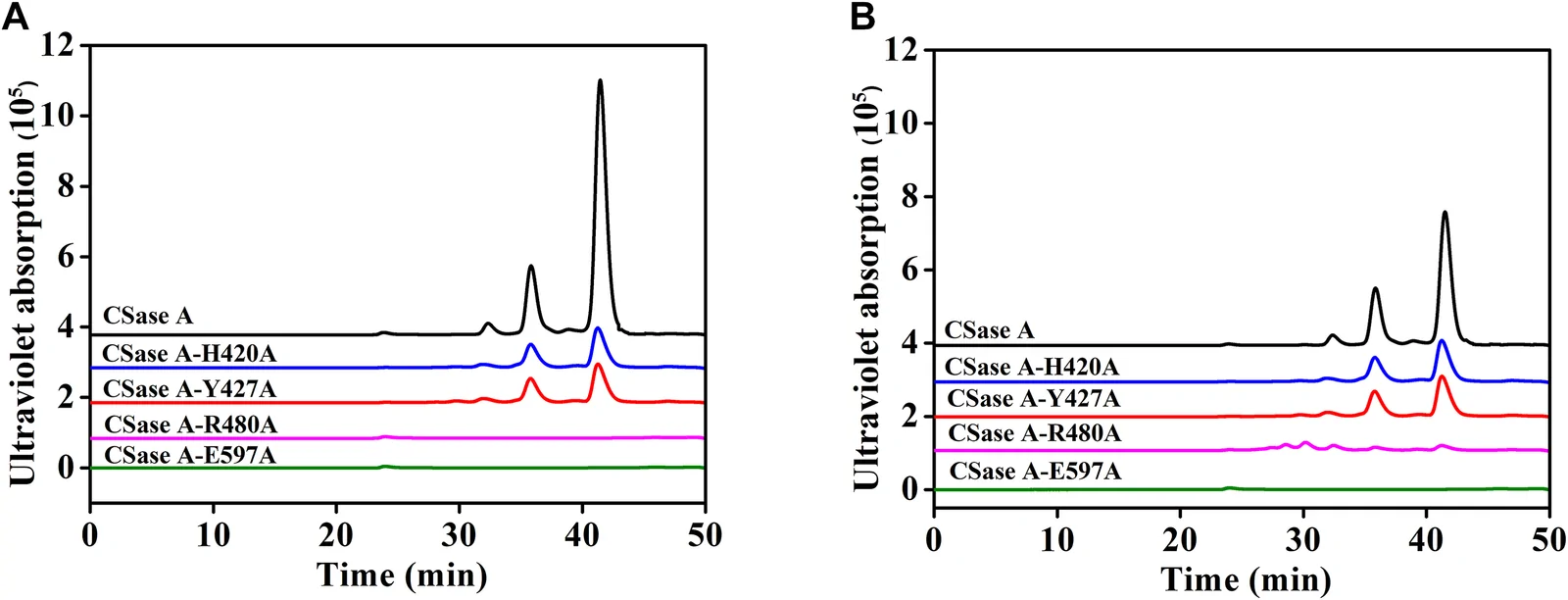
**Activity analysis of CSase A mutants.** The crude enzymes of CSase A and its four mutants CSase A-H420A, CSase A-Y427A, CSase A-R480A, and CSase A-E579A were utilized to degrade the two substrates CS-A (*A*) and DS (*B*). The degradation products were analyzed by gel filtration chromatography column. CSase A, chondroitinase A; CS, chondroitin sulfate; DS, dermatan sulfate.

## Discussion

The discovery and characterization of CSase A represent a significant advancement in the field of CS/DS lyases. This enzyme shows relatively low sequence similarity to previously identified GAG lyases (<30%) and exhibits unique biochemical properties that differentiate it from other CS/DS lyases. Specifically, CSase A displays pronounced and previously unreported selectivity for cleaving glycosidic bonds linked to the 4-*O*-sulfated A/iA unit within CS/DS chains. To our knowledge, this is the first functional characterization of a CSase A-type enzyme.

In this study, the biochemical and degradative properties of CSase A were systematically investigated. The enzyme belongs to the PL8 family and clusters phylogenetically with known CSase ABC enzymes. Preliminary degradation assays confirmed that, like typical CSase ABC enzymes, it can degrade a broad range of CS types, DS, and HA. Notably, however, its amino acid sequence shares less than 30% identity with any previously characterized CSases, suggesting substantial structural and evolutionary divergence. Time-course analysis of the disaccharide products generated during digestion revealed a sequential degradation pattern: disaccharides containing A units appeared first, followed by nonsulfated O units, whereas disaccharides containing C units were barely detected throughout the digestion process. This stepwise product profile suggests that the enzyme preferentially targets 4-*O*-sulfated regions while exhibiting reduced activity toward nonsulfated domains. Enzyme activity assays further underscored its remarkable substrate selectivity. CSase A exhibited substantially higher specific activity toward CS-A and DS, both rich in A/iA units, compared to its activity toward CS-C (rich in C units), CS-D (containing both C and D units), and CS-E (particularly rich in 4,6-*O*-disulfated E units). Although the enzyme was also capable of degrading HA—which possesses an alternating GlcA-GlcNAc backbone but lacks sulfate groups—its efficiency toward this substrate was notably lower than that toward CS-A. This substrate preference profile, combined with the observed disaccharide product patterns, establishes CSase A as a unique enzymatic tool with strict specificity for 4-*O*-sulfated motifs, distinguishing it from all previously characterized CSases and providing a valuable probe for dissecting the structure-function relationships of A/iA unit-rich domains in CS/DS biology.

To define the true substrate range and selectivity boundaries of the enzyme, we performed exhaustive degradation analyses. Comprehensive substrate degradation profiling revealed that CSase A depolymerizes CS, DS, and HA primarily into oligosaccharides. Disaccharide composition analysis of the final CS-A digest confirmed a distinct partition between the resistant oligosaccharides and the released disaccharides: ΔC (representing 6-*O*-sulfated GalNAc) was highly enriched in the tetrasaccharide (62.74%) and hexasaccharide (88.21%) fractions, whereas ΔA (containing 4-*O*-sulfated GalNAc) dominated the disaccharide products (93.06%) (Table 2). This progressive enrichment of 6-*O*-sulfated units with increasing oligosaccharide chain length indicates that CSase A preferentially cleaves glycosidic bonds adjacent to 4-*O*-sulfated GalNAc residues while exhibiting resistance toward regions containing 6-*O*-sulfated motifs. Furthermore, analysis of the disaccharide products generated from structurally diverse substrates revealed that, regardless of the complexity of the CS/DS substrate, the overwhelming majority of the resulting disaccharides were ΔA units. Structural analysis of the degradation products established the molecular basis for this selectivity, confirming that the enzyme recognizes the sulfation pattern at the GalNAc C4 position as a key determinant for catalytic cleavage. Exhaustive digestion of CS-A generated resistant tetrasaccharides (ΔC-C and ΔA-C) and resistant hexasaccharides (ΔC-C-C and ΔA-C-C), which terminate with a 6-*O*-sulfated GalNAc (C unit) at their reducing ends (Fig. 6). This finding indicates that 6-*O*-sulfation at the reducing-end GalNAc acts as a structural barrier to cleavage by CSase A. Definitive validation using defined, synthetically accessible tetrasaccharide substrates showed that CSase A cleaved only those bearing reducing-end A and O unit (*e.g.*, ΔE-A, ΔD-O), while tetrasaccharides with reducing-end C, D, E, or iB units remained intact (Fig. 7). These findings imply that sulfation patterns other than 4-*O*-sulfation (such as 6-*O*- or 2-*O*-sulfation) may sterically or electrostatically hinder substrate binding or proton transfer at the catalytic center, thereby suppressing cleavage activity. Collectively, these results demonstrate that CSase A strictly requires a nonsulfated or 4-*O*-sulfated GalNAc at the −1 subsite (the reducing-end position after cleavage) for efficient catalysis. This specificity designates CSase A as the first strict A unit-specific endolyase, a functional profile that clearly distinguishes it from all previously characterized CSases (including the broad-specificity ABC type, the CS-specific AC type, the DS-specific B type, and the 6-*O*-sulfate-preferring C type) (25, 28, 29, 31, 32, 33, 35, 40).

Based on homology modeling and site-directed mutagenesis, the catalytic mechanism of CSase A appears to depend critically on four conserved residues Glu-579, Arg-480, Tyr-427, and His-420. The complete loss of activity in the H420A mutant suggests that His-420 likely serves as an essential proton donor or acceptor. The substrate-specific behavior observed in the Y427A mutant, complete loss of activity toward CS-A but not DS, implies a key role for Tyr-427 in substrate recognition and specificity. Partial inactivation resulting from the E579A and R480A mutations indicates supporting roles for these residues in transition-state stabilization and/or substrate alignment. Together with its lack of metal ion dependence, these findings suggest that CSase A likely employs a Tyr–His-mediated acid–base catalytic mechanism. Further structural studies are necessary to elucidate the precise proton transfer network and substrate recognition machinery.

CS-A, enriched in A unit, plays critical roles in diverse physiological and pathological contexts. Notably, it enhances the viability, proliferation, migration, and invasion capabilities of glioma cells, suggesting its potential as a therapeutic target for glioma treatment (41). In osteoarthritis, where CS constitutes over 80% of GAGs in articular cartilage, a significant downregulation of 4-*O*-sulfated CS is observed, implicating its involvement in cartilage degeneration and highlighting its potential as a diagnostic biomarker as well as its importance in cartilage regeneration (42). Furthermore, in placental malaria, the VAR2CSA protein secreted by *Plasmodium* *falciparum*-infected red blood cells specifically binds to regions rich in A unit on the surface of syncytiotrophoblasts, facilitating erythrocyte adhesion to the vascular endothelium and thereby contributing to the pathogenesis of placental malaria (18, 19, 20). However, the extreme structural complexity of CS/DS including variations in sulfation patterns (at C-4/C-6 of GalNAc and C-2 of GlcA/IdoA), epimerization extent (GlcA to IdoA), and heterogeneous modification degrees poses a significant challenge to the precise elucidation of functional oligosaccharide sequences. Conventional chemical and physical degradation methods lack the requisite site-specificity, resulting in inefficient preparation of bioactive oligosaccharides and greatly limiting the development of targeted glycan-based therapeutics and in-depth mechanistic studies of related diseases. In this context, the discovery and application of CSase A, which specifically cleaves 4-*O*-sulfated A unit/iA unit-rich regions, offer a transformative tool. By enabling targeted digestion of these functionally significant domains, CSase A facilitates the production of well-defined oligosaccharides critical for probing structure-function relationships. It also allows for the specific detection and quantification of A unit-containing segments in complex CS/DS samples. Moreover, this enzymatic specificity supports the selective preparation of functional oligosaccharides with distinct sulfation patterns, thereby accelerating the development of novel carbohydrate-based drugs and providing new insights into the mechanisms underlying diseases associated with 4-*O*-sulfated CS.

In conclusion, CSase A has been identified and functionally characterized as the first member of a novel CSase subfamily, CSase A, exhibiting strict and unprecedented specificity toward A/iA unit within CS/DS chains. Its unique cleavage pattern, structural features, and catalytic mechanism distinguish it from all previously known CSase classes (ABC, AC, B, and C). The discovery of CSase A not only fills a fundamental gap in the repertoire of GAG-degrading enzymes but also provides a powerful and selective tool for the precise dissection of CS/DS structure-function relationships, preparation of biologically active oligosaccharides, and the development of glycan-based diagnostic and therapeutic strategies targeting A unit-mediated biological processes and diseases.

## Experimental procedures

### Materials

The strains and plasmids used in this study are listed in Table 3. SDS, proteinase K, PrimeSTAR HS DNA polymerase, restriction endonuclease, and other genetic engineering enzymes were purchased from Takara Inc. Standard unsaturated disaccharides, CS-A from whale cartilage, CS-C from shark cartilage, CS-D from shark fin, CS-E from squid cartilage, and DS, Hep, and HS from porcine intestinal mucosa were purchased from GlycoSciTech Co, Ltd. Sodium cyanoborohydride (NaBH_3_CN), 2-aminobenzamide (2-AB), Alg from brown alga and CSase ABC (EC 4.2.2.4) were purchased from Sigma-Aldrich. All other chemicals and reagents were of the highest quality available. CS/DS-derived structurally determined tetrasaccharides and HRDS were prepared by our laboratory (27, 38, 43).

**Table 3:** Bacterial strains, plasmids, and primers used for sequencing in the present study

Strain or plasmid	Description	Source
*E. coli* BL21 (DE3)	F-, ompT, hsdSB (rB-, mB-), dcm, gal, λ (DE3), pLysS, Cmr	Novagen
Plasmid		
pET30a	Expression vector; Kan^r^	Novagen
pET30a-CSaseA	pET30a carrying an amplified *Nde* I-*Xho* I fragment encoding the recombinant protein of CSaseA fused with a His6 tag at the C terminus.	This study
Sequencing primers		
CSase A	F: 5′-GCCATATGGCCCAATTGATCGGCTTTG-3′	
CSase A	R: 5′-CCCTCGACTTATTCTCCATCAACATC-3′	
CSase A-H420	F: 5′-GTTTCATGGTGGGGGCTTC-3′	
CSase A-H420	R: 5′-GAAGCCCCCATGATGAAAC-3′	
CSase A-Y427	F: 5′-CAGGCTACACGGCGGGCGTG-3′	
CSase A-Y427	R: 5′-CACGCCCGCCGTGTAGCCTG-3′	
CSase A-R480	F: 5′-CTCAGGTGGGCATCCGTTTGG-3′	
CSase A-R480	R: 5′-CCAAACGGATGCCCACCTGAG-3′	
CSase A-E579	F: 5′-GAAGCGGGATCTACACTAAAG-3′	
CSase A-E579	R: 5′-CATAAATGGCAGAACCCCATACATCAGTCGTATATC-3′	

### Identification and sequence analyses of CSase A genes and proteins

Sequences from the metagenome of sediments near the Haima cold seep were subjected to the following analysis. Promoter motifs of the 5′-flanking DNA region upstream of the ORF were identified using Primer Premier version 5.0 (PREMIER Biosoft International, Palo Alto, Calif) and the Promoter 2.0 Prediction Server (http://www.cbs.dtu.dk/services/Promoter/). The G + C content (G + C%) of the gene was calculated using Bio-Edit version 7.0.5.3. The sequence similarity analysis of the protein sequence was performed using the BLASTp algorithm online (https://blast.ncbi.nlm.nih.gov/Blast.cgi). Sequence alignment and phylogenetic analysis were performed using BioEdit software (version 7.2.5) and MEGA version 5.05. Secretion signal peptides and their types were identified using the SignalP 4.0 server and LipoP 1.0 server, respectively. Protein modules and domains were identified using the Simple Modular Architecture Research Tool, Pfam database (SMART, http://smart.embl-heidelberg.de/), and Carbohydrate-Active enZYme database (CAZy) (CAZy; http://www.cazy.org/CAZY/).

### Heterologous expression of the CSase A gene

To express CSase A (excluding the N-terminal signal peptide) in *E. coli* strains, the full-length gene excluding signal peptide was amplified using high fidelity PrimeSTAR HS DNA polymerase (Takara Inc) and primer pairs (as listed in Table 3) containing restriction enzyme sites (underlined). These sites were designed to match the insertion sites of the expression vectors pET-22b(+) and pET-30a(+) (Novagen). The gel-purified PCR products were cloned into these expression vectors, and then expression vectors from pET-22b(+) (pE22b-rCSase A) and pET-30a(+) (pE30a-rCSase A) were transformed into *E. coli* BL21(DE3) cells. The integrity of the nucleotide sequences of all constructed plasmids was confirmed *via* DNA sequencing. *E. coli* cells harboring an expression vector were initially cultured in LB broth. When the optical density at 600 nm (A_600_) reached 0.8 to 1.0, expression of the target protein was induced by adding IPTG to a final concentration of 0.01 mM. After a continual cultivation for an additional 24 h at 16 °C, cells were harvested by centrifugation at 6000*g* for 15 min, washed twice with ice-cold Buffer A (50 mM Tris-HCl, 150 mM NaCl, pH 8.0), and resuspended in Buffer A. Cell disruption was performed by sonication on ice (50 cycles of 5 s pulse). The lysate was centrifuged at 15,000*g* for 30 min, and the supernatant was collected for subsequent purification of soluble target protein.

### Purification of recombinant protein rCSase A

To purify the rCSase A protein, the supernatant containing soluble native enzyme was loaded onto a column packed with Ni Sepharose 6 Fast Flow (GE Healthcare), then the column was washed with buffer A containing 50 mM imidazole to remove impurities. Bound rCSase A was subsequently eluted using a linear imidazole gradient (50–250 mM) in Buffer A. Protein purity was assessed by SDS-PAGE performed on 13.2% polyacrylamide gels according to Sambrook and Russell (44), with protein bands visualized by Coomassie Brilliant Blue R-250 staining. Protein concentrations were determined using the Folin-Lowry method (45).

### Gel filtration chromatography

Enzymatic digests generated by rCSase A were analyzed using gel filtration chromatography on a Superdex peptide 10/300 Gl column (Cytiva). The mobile phase was 0.20 M NH_4_HCO_3_ at a flow rate of 0.4 ml/min, and the eluted fractions were monitored at 232 nm using a UV detector. Data acquisition and analysis were performed using the software LCsolution version 1.25.

### Disaccharide composition analysis

Each sample was digested with CSase ABC (Sigma-Aldrich) and labeled with 2-AB and NaBH_3_CN reagents as described by Bigge *et al.* (46). And then the products were individually analyzed by anion-exchange HPLC on a YMC-Pack PA-G column (YMC), eluted by linear gradient of NaH_2_PO_4_ (16 mM to 460 mM) at a flow rate of 1 ml/min over 60 min. Monitoring through fluorescence detector with excitation and emission wavelengths of 330 nm and 420 nm, standard CS/DS unsaturated disaccharides were applied for qualitative and quantitative purposes. The online monitoring and data analysis (such as molar ratio determination) were performed using the software LC solution version 1.25.

### Substrate specificity assay of rCSase A

To determine the substrate specificity of rCSase A, the enzyme was incubated with various polysaccharide substrates. Stock solutions (10 mg/ml in deionized water) of Alg, CS-A, CS-C, CS-D, CS-E, DS, Hep, HS, and HA were prepared. The disaccharide assay of different CS/DS are shown in Table S1. For each assay, 100 μl of polysaccharide stock solution was mixed with 200 μl of 250 mM NaH_2_PO_4_-Na_2_HPO_4_ buffer (pH 7.0), 600 μl of deionized water, and 100 μl of appropriately diluted rCSase A (100 μg) enzyme solution. Reactions were incubated at 37 °C for 12 h. Following incubation, samples were heated in 85 °C for 10 min to terminate the reaction, then immediately cooled on ice for 10 min. After being centrifuged at 15,000 × *g* for 15 min, the supernatant was collected and analyzed by the absorbance at 232 nm (indicative of unsaturated products) (43).

To further determine the structural characteristics of the complete degradation products, CS-A, CS-C, CS-D, CS-E, DS and HA (100 μg each) were separately digested with 10 μg of rCSase A at 10 °C for 48 h in 50 mM NaH_2_PO_4_-Na_2_HPO_4_ buffer (pH 6.0). Fresh enzyme (5 μg) was supplemented at 24 h during the incubation. The reaction was terminated by heat inactivation. The resulting products (20 μg) were collected by centrifugation and analyzed by gel-filtration chromatography. To determine whether the enzyme exhibits any preference, the disaccharide products were collected and analyzed following the same procedure as described for “Disaccharide composition analysis,” with the only difference being that CSase ABC digestion was omitted.

### Analysis of optimal conditions and activity assay for rCSase A

The optimal reaction conditions for rCSase A were determined using CS-A for temperature optimization and CS-A, CS-C, and HA for pH-dependent activity measurements. Optimal temperature was assessed by incubating reaction mixtures (1 ml) containing 50 mM NaH_2_PO_4_-Na_2_HPO_4_ buffer (pH 7.0), 1 mg CS-A, and 10 μl of appropriately diluted rCSase A (100 μg) at 0 to 70 °C for 30 min. The effect of pH was then examined at 10 °C using CS-A, CS-C, and HA in 50 mM NaAc-HAc (pH 5.0–6.0), 50 mM NaH_2_PO_4_-Na_2_HPO_4_ (pH 6.0–8.0), and 50 mM Tris-HCl (pH 7.0–10.0), in a total volume of 1 ml. Metal ions and chelators (final concentration, 5 mM) were tested using CS-A under the optimal conditions. Thermal stability was evaluated by preincubating rCSase A in 50 mM NaH_2_PO_4_-Na_2_HPO_4_ buffer (pH 6.0) at 0 to 70 °C for 0 to 24 h, followed by residual activity measurement under optimal conditions using CS-A. For freeze-thaw stability assays, each cycle consisted of freezing at −20 °C for 2 h and thawing at RT for 10 min; residual activity was measured after 0 to 6 cycles. To identify an MS-compatible reaction system, activity toward CS-A was measured at pH 6.0 and 10 °C in 50 mM NaH_2_PO_4_-Na_2_HPO_4_ buffer (control), without an added buffer after direct pH adjustment to 6.0, or in 50 mM ammonium acetate buffer (pH 6.0). All reactions were performed in triplicate, and activity was determined by measuring the increase in absorbance at 232 nm (27). For data normalization, the maximal activity observed for CS-A under its optimal conditions (temperature and pH) was defined as 100%. For metal ion, chelators and freeze-thaw assays, activities measured without metal ions and chelators or before any freeze-thaw cycle, respectively, were set as 100%. Based on these optimizations, all subsequent enzymatic digestions (unless otherwise specified) were carried out at 10 °C in 50 mM NaH_2_PO_4_-Na_2_HPO_4_ buffer (pH 6.0).

The enzymatic activity of rCSase A was determined under their respective optimal conditions using CS-A, CS-C, CS-D, CS-E, DS, and HA as substrates, based on the principle of unsaturated product detection at 232 nm (43). One unit (U) of activity was defined as the amount of enzyme required to produce 1 μmol of unsaturated bonds per minute under the specified conditions.

### Time-course degradation experiment

The degradation pattern of rCSase A was examined using CS-A as the substrate. CS-A (1 mg) was digested with rCSase A (100 μg) in a 1 ml reaction at 10 °C. To obtain a complete degradation profile, aliquots (100 μl) were taken at 0, 1, 2, 4, 8, 12, 24, and 48 h, and fresh enzyme (20 μg) was supplemented every 12 h during incubation. Each aliquot was heated at 85 °C for 10 min, cooled to 4 °C, and centrifuged (15,000 × *g*, 30 min). The supernatant (10 μl) was filtered and analyzed by gel-filtration chromatography on a Superdex peptide 10/300 Gl column under previously described conditions, with online detection at 232 nm to monitor the time-dependent generation of oligosaccharide products. The product disaccharides were also isolated and analyzed to assess the degradation tendency of CSase A, using the same method as for “Disaccharide composition analysis” but without CSase ABC treatment. The remaining 200 μl 48 h digest was processed in the same manner for subsequent structural analysis of resistant oligosaccharides.

### Structure analysis of rCSase A-resistant tetrasaccharides and hexasaccharides derived from CS-A

To further define the structural features of the exhaustive CS-A digestion products, the 48 h digest was fractionated by gel-filtration chromatography to isolate the tetrasaccharide and hexasaccharide fractions. The collected material was lyophilized three times to remove residual NH_4_HCO_3_ for further identification. The disaccharide composition of the fractions was determined as described under “Disaccharide composition analysis”. Subsequently, the resistant tetrasaccharide and hexasaccharide fractions were separately isolated by anion-exchange HPLC on a YMC-Pack PA-G column with online monitoring at 232 nm. The tetrasaccharide fraction was eluted with a linear gradient of 16 to 460 mM NaH_2_PO_4_ over 60 min (1 ml/min), whereas the hexasaccharide fraction was eluted with a linear gradient of 0 to 700 mM NaH_2_PO_4_ over 60 min (1 ml/min). The major peaks were collected and desalted on a Superdex peptide column as described previously. The resulting subfractions 1 and 2 were sequenced at a low picomole level by CSase and HPLC (38). For tetrasaccharide, to analyze the disaccharide composition of the resistant tetrasaccharides, an aliquot (5 pmol) of the tetrasaccharide subfraction was digested with CSase ABC (Sigma-Aldrich) and labeled with the 2-AB and NaBH_3_CN reagents. Secondly, to identify the reducing end, the subfractions (5 pmol) used for sequencing were labeled with 2-AB and then treated with HCDLase from *Vibrio* sp. FC509 (47). All of these preparations were individually analyzed by anion-exchange HPLC on a YMC-Pack PA-G column using a fluorescence detector as in “Disaccharide composition analysis”. To further determine the molecular weight and structural characteristics of the CSase A-resistant tetrasaccharides, the two major subfractions (subfraction 1 and 2) were analyzed by electrospray ionization MS on an IT-TOF hybrid mass spectrometer (LCMS-IT-TOF) with negative-ionization mode. The acquisition range was set to m/z: 200 to 1200.

For hexasaccharide sequencing, in addition to the reducing end identification using the same HCDLase method, the nonreducing end disaccharide was determined as follows: an aliquot was first labeled with 2-AB (reducing-end labeling), then digested with CSase ABC, and the resulting products (where the nonreducing terminal disaccharide now exposes a free reducing end) were subjected to a second round of 2-AB labeling. All labeled preparations were individually analyzed by anion-exchange HPLC on a YMC-Pack PA-G column with fluorescence detection as described in “Disaccharide composition analysis”.

### Analysis of CSase A degradation specificity using defined tetrasaccharide standards

To further investigate the structural determinants of CSase A substrate specificity, a panel of defined unsaturated tetrasaccharide standards were utilized as substrates. The standards included ΔE-A, ΔE-C, ΔE-E, ΔD-A, ΔA-D, and ΔB-iB. Each specific tetrasaccharide standard (10 pmol) was treated under optimal conditions to allow for enzymatic digestion. Following incubation, the products were analyzed by anion-exchange HPLC on a YMC-Pack PA-G column, with detection carried out using a fluorescence detector. For most tetrasaccharide standards, the elution gradients were those described in the “Disaccharide composition analysis” section; modified gradients were used when required for adequate separation. To determine whether 4-*O*-sulfation of the reducing-end GalNAc is required for cleavage, ΔC-A and ΔD-A were first treated with a 4-*O*-sulfatase (48) to remove the 4-*O*-sulfate group, converting the reducing-end A unit to an unsulfated O unit. The resulting products were then incubated with CSase A and analyzed by the same anion-exchange HPLC method. In addition, nonsulfated chondroitin was incubated with CSase A under the optimal conditions, and the resulting products were analyzed by size-exclusion chromatography on a Superdex Peptide 10/300 Gl column with monitoring at 232 nm.

### Structural modeling and mutation analysis of CSase A

To investigate the functions of the specific amino acid residues in the HA/CS-degrading activities of CSase A, its protein sequence was submitted to the SWISS-MODEL server (https://swissmodel.expasy.org/) for automated homology modeling. Homology modeling was first performed to predict the three-dimensional structure of CSase A using the structure (PDB ID: 1HN0) of CSase ABC I from *P. vulgaris* as a template. The computational model showed that the His-420, Tyr-427, Arg-480, and Glu-579 residues may affect the interaction between the enzyme and substrate. To determine the role of these residues, site-directed mutagenesis was performed on the pET-30a-CSase A plasmid template to substitute each residue with alanine (H420A, Y427A, R480A, E579A) using rapid PCR amplification with high fidelity DNA polymerase of PrimeSTAR MAX Premix (Takara Inc.) to form a series of gene mutation products. Corresponding primer pairs are listed in Table 3. Amplified gene products were individually phosphorylated at the 5′-end, circulated, and transformed into *E. coli* BL21(DE3) cells, and protein expression was then induced. GAG-degrading activities were measured as described previously.

## Data availability

All data supporting the findings of this study are available within the paper (and its supporting information files). All relevant data generated during this study or analyzed in this published article are available from the corresponding author on reasonable request. Source data are provided with this paper.

## Supporting information

This article contains supporting information.

## Conflict interest

The authors declare that they have no conflicts of interest with the contents of this article.
