# Supplementary material for: Discovery and characterization of chondroitinase A with specificity for 4-O-sulfated GalNAc in chondroitin sulfate/dermatan sulfate
Source: J Biol Chem. 2026 Sep 22;302(11):113594. doi: 10.1016/j.jbc.2026.113594 (PMC13696812; doi:10.1016/j.jbc.2026.113594)
Supplement: Supplementary file 1 [file mmc1.docx]

**Discovery and characterization of Chondroitinase A with specificity for 4‑*O*‑sulfated GalNAc in chondroitin sulfate/dermatan sulfate**

Wenshuang Wang^1^, Haixin Yi^1^, Ping’en Zi^1^, Xu Wang^1^, Rouyi Zou^1^, Yu-zhong Zhang^1^ and Fuchuan Li^1^*.

**Author’s Affiliation**

^1^ National Glycoengineering Research Center, State Key Laboratory of Microbial Technology, Shandong Center of Technology Innovation for Carbohydrate, Shandong Key Laboratory of Carbohydrate and Carbohydrate-conjugate Drugs, Shandong University, 72 Binhai Rd, Qingdao, 266237, People’s Republic of China.

**Corresponding author**

*(F. Li) Email: fuchuanli@sdu.edu.cn. Tel: +86-532-58631406 Fax: +86-532-58631405.

**Fig. S1** Isolation and purification of tetrasaccharides after complete degradation of CS-A by CSase A

**Fig. S2** Time-of-flight mass spectrometry analysis of resistant tetrasaccharide subfractions.

The final tetrasaccharide products from CS-A were isolated and purified. Two fractions, ΔC-C (A) and ΔA-C (B), were analyzed by mass spectrometry. inten., intensity.

**Fig. S3** Isolation and purification of hexasaccharides after complete degradation of CS-A by CSase A.

**Fig. S4** Detection of disaccharide fragments after complete degradation of intact chondroitin by CSase A. 1, nonsulfated disaccharide

**Table S1** Analysis of disaccharide composition of different substrates.

| CS/DS | ΔO | ΔC | ΔA | ΔB | ΔD | ΔE |
| --- | --- | --- | --- | --- | --- | --- |
|  | Mol % | | | | | |
| CS-A | 6.52 | 11.80 | 81.68 | ND | ND | ND |
| CS-C | 0.72 | 54.07 | 27.18 | ND | 18.03 | ND |
| CS-D | 0.45 | 52.44 | 14.70 | ND | 32.41 | ND |
| CS-E | 1.32 | 8.43 | 30.06 | ND | ND | 60.19 |
| DS | 2.50 | ND | 85.71 | 11.79 | ND | ND |
| HRDS | 3.40 | ND | 22.35 | 74.25 | ND | ND |

Notes: Mol %: The molar ratio of disaccharide units was calculated based on their peak areas;

ND: not detected.

**Table S2** Analysis of collected disaccharide after degradation by CSase A

| CS/DS | ΔO | ΔC | ΔA | ΔB | ΔD | ΔE |
| --- | --- | --- | --- | --- | --- | --- |
|  | Mol % | | | | | |
| CS-A | 3.52 | ND | 96.48 | ND | ND | ND |
| CS-C | 1.19 | ND | 98.81 | ND | ND | ND |
| CS-D | 2.51 | ND | 97.49 | ND | ND | ND |
| CS-E | 1.85 | ND | 98.15 | ND | ND | ND |
| DS | 1.87 | ND | 98.13 | ND | ND | ND |

Notes: Mol %: The molar ratio of disaccharide units was calculated based on their peak areas;

ND: not detected.

**Table S3** Analysis of disaccharide proportions collected after time‑course degradation of CS‑A by CSase A

| Time | ΔO | ΔC | ΔA | ΔB | ΔD | ΔE |
| --- | --- | --- | --- | --- | --- | --- |
|  | Mol % | | | | | |
| 2 h | ND | ND | 100 | ND | ND | ND |
| 4 h | 0.52 | ND | 99.48 | ND | ND | ND |
| 12 h | 3.45 | ND | 96.55 | ND | ND | ND |
| 24 h | 6.87 | ND | 93.13 | ND | ND | ND |
| 48h | 6.94 | ND | 93.06 | ND | ND | ND |

Notes: Mol %: The molar ratio of disaccharide units was calculated based on their peak areas;

ND: not detected.
